# Blood levels of adiponectin and IL-1Ra distinguish type 3c from type 2 diabetes: Implications for earlier pancreatic cancer detection in new-onset diabetes

**DOI:** 10.1016/j.ebiom.2021.103802

**Published:** 2022-01-03

**Authors:** Lucy Oldfield, Anthony Evans, Rohith Gopala Rao, Claire Jenkinson, Tejpal Purewal, Eftychia E. Psarelli, Usha Menon, John F. Timms, Stephen P. Pereira, Paula Ghaneh, William Greenhalf, Christopher Halloran, Eithne Costello

**Affiliations:** a Department of Molecular and Clinical Cancer Medicine, University of Liverpool, UK; b Department of Diabetes and Endocrinology, Royal Liverpool University Hospital, UK; c Institute of Clinical Trials and Methodology, University College London, UK; d Women's Cancer, Institute for Women's Health, University College London, UK; e Institute for Liver and Digestive Health, University College London, UK

**Keywords:** Pancreatic cancer, Early detection, Type 3c diabetes, Blood biomarkers, Adiponectin, IL-1Ra

## Abstract

**Background:**

Screening for pancreatic ductal adenocarcinoma (PDAC) in populations at high risk is recommended. Individuals with new-onset type 2 diabetes mellitus (NOD) are the largest high-risk group for PDAC. To facilitate screening, we sought biomarkers capable of stratifying NOD subjects into those with type 2 diabetes mellitus (T2DM) and those with the less prevalent PDAC-related diabetes (PDAC-DM), a form of type 3c DM commonly misdiagnosed as T2DM.

**Methods:**

Using mass spectrometry- and immunoassay-based methodologies in a multi-stage analysis of independent sample sets (n=443 samples), blood levels of 264 proteins were considered using Ingenuity Pathway Analysis, literature review and targeted training and validation.

**Findings:**

Of 30 candidate biomarkers evaluated in up to four independent patient sets, 12 showed statistically significant differences in levels between PDAC-DM and T2DM. The combination of adiponectin and interleukin-1 receptor antagonist (IL-1Ra) showed strong diagnostic potential, (AUC of 0.91; 95% CI: 0.84-0.99) for the distinction of T3cDM from T2DM.

**Interpretation:**

Adiponectin and IL-1Ra warrant further consideration for use in screening for PDAC in individuals newly-diagnosed with T2DM.

**Funding:**

North West Cancer Research, UK, Cancer Research UK, Pancreatic Cancer Action, UK.

**Research in context**

**Evidence before this study**

New-onset diabetes mellitus (NOD) is associated with an increased risk of pancreatic cancer. Screening of high-risk groups for PDAC is recommended. However, since T2DM is common it is not feasible, with current modalities, to undertake PDAC screening of NOD without further enriching this population.

Diabetes mellitus (DM) occurring secondary to pancreatic disease, known as type 3c DM (T3cDM), is frequently misdiagnosed as type 2 DM (T2DM). Biomarkers that distinguish T3cDM from T2DM could help enrich the NOD population for PDAC, facilitating screening. No such biomarkers exist.

**Added value of this study**

Having considered 264 blood proteins, and evaluated 30 through training and/or validation using independent sample sets, 12 proteins showed significantly different levels in T3cDM versus T2DM, regardless of whether the T2DM was new-onset or long-standing.

The combination of adiponectin and interleukin-1 receptor antagonist (IL-1Ra) showed strong diagnostic potential for the distinction of T3cDM from T2DM.

**Implications of all the available evidence**

Our results provide a basis for biomarker-driven stratification of individuals with NOD into high or low risk for PDAC, based on whether diabetes is T3cDM or T2DM. Adiponectin and IL-1Ra warrant validation in bespoke pre-diagnostic NOD cohorts for this purpose.

Alt-text: Unlabelled box

## Introduction

Pancreatic ductal adenocarcinoma (PDAC) is a leading cause of cancer-related deaths worldwide.^1^ The disease is most often diagnosed at an advanced stage, contributing to the low overall five-year survival of 5-10%. Screening could facilitate earlier detection.^2^ However, PDAC is uncommon, occurring at an annual incidence of 5-17 per 100,000 population worldwide. Consequently, only screening tests with specificities close to 100% would avoid large numbers of false positives. CA19-9, the only biomarker routinely used for the clinical management of PDAC, suffers from poor specificity and cannot be used as a stand-alone diagnostic for screening.

An estimated 10% of PDAC patients have a family history of the disease and individuals in such families are offered screening.^3^ For the remaining 90% of sporadic PDAC cases, no suitable screening test currently exists and new approaches to early detection are much needed. Recently it has become evident that individuals over the age of 50 with a new diagnosis of diabetes mellitus (DM) are a high-risk group for PDAC.^4^ At the point of diagnosis of PDAC, ∼80% of patients have abnormal fasting glucose or glucose intolerance.^5^ The high rate of hyperglycaemia occurring in PDAC is not observed in other common cancers, such as lung, breast, prostate, and colorectal, where the prevalence of DM closely matches that of the general population.^6^ PDAC-related hyperglycaemia first occurs up to three years prior to PDAC diagnosis, with diabetes observed 12 to 6 months before diagnosis.^7^^,^^8^ Thus, the onset of DM may be considered a paraneoplastic ‘symptom’ of PDAC and individuals with new-onset DM (NOD) the highest risk group for PDAC.

It is not feasible to screen all individuals with NOD for PDAC, and criteria that enrich for a subpopulation of NOD most at risk are sought.^9^ A subgroup of individuals diagnosed with diabetes actually have DM secondary to pancreatic disease, frequently termed type 3c diabetes mellitus (T3cDM; comprising both PDAC- and chronic pancreatitis (CP)-related diabetes, as well as other aetiologies).^10^^,^^11^ Difficulties associated with accurate classification of diabetes in clinical practice and under-diagnosis of diabetes mean that the actual prevalence of T3cDM amongst those diagnosed with diabetes is not known. Depending on study design, different estimates are reported, ranging from 1.8 – 9.2%.^10^^,^^12^^,^^13^ A working estimate of 4-5% has been suggested as reasonable.^7^^,^^14^ Of all T3cDM cases, a significant proportion have PDAC-related diabetes (PDAC-DM), reported variously as 8.1%,^13^ and 31%.^15^ Identifying biomarkers that distinguish new-onset T3cDM from new-onset type 2 diabetes (T2DM), would enable screening of the T3cDM group, facilitating earlier diagnosis of PDAC. Here we describe a multi-stage programme of work, specifically designed to identify protein biomarkers for T3cDM, with candidate biomarkers evaluated through sequential independent sample sets. This process culminated in two candidates, adiponectin and interleukin-1 receptor antagonist (IL-1Ra), capable of distinguishing T3cDM from T2DM with a high degree of accuracy.

## Methods

### Patient groups

This study followed STARD guidelines. Blood samples from individuals with PDAC, chronic pancreatitis (both pre-surgical), DM (following referral from diabetes clinics and primary care centres) and healthy subjects were collected at the Royal Liverpool University Hospital (RLUH) and the University of Liverpool between 2006 and 2017. Diabetes was defined as HbA1c ≥48 mmol/mol, or confirmation of diabetes medication, and categorised as long-standing diabetes (>3 years post-diagnosis of DM) or new-onset diabetes (≤3 years post-diagnosis of DM). Pre-diagnostic blood was obtained from women recruited to the UK Collaborative Trial of Ovarian Cancer Screening (UKCTOCS) study between 2001 and 2005.

### Ethics

This study was approved by the Health Research Authority with favourable opinions from the North West – Greater Manchester East and the London – South East Research Ethics Committees (Ethics Identifiers: 11/NW/0083 and 16/LO/1630, respectively). All participants gave written consent after having an opportunity to discuss the research and before any data or samples were collected. The UKCTOCS study was approved by the UK North West Multicentre Research Ethics Committee (North West MREC 00/8/34) and the London – Bentham Research Ethics committee (Ethics Identifier: 05/Q0505/57). All participants gave written consent.

### Cohorts

Four independent sample sets were analysed. Set 1, comprising a nested set (n=60) of a larger retrospective cohort described previously,^16^ was used for the analysis of adiponectin levels in healthy individuals (n=20) versus individuals with histologically confirmed PDAC (n=40, 12 with diagnosed diabetes and 28 without diagnosed diabetes; Supplementary Table S1). Set 2 (n=137, Table 1a), used for targeted training of diabetes-associated proteins, included 80 individuals with histologically confirmed PDAC (41 with diagnosed diabetes and 41 without diagnosed diabetes), 20 individuals with chronic pancreatitis (10 with diagnosed diabetes and 10 without diagnosed diabetes), 20 individuals with long-standing (>3 years post-diagnosis) T2DM and 15 healthy subjects. Set 3 (n=175; Table 1b; Supplementary Table S2), used to validate candidate markers in individuals with new-onset T2DM (≤3 years post-diagnosis), consisted of 78 individuals with histologically confirmed PDAC (37 with diagnosed diabetes and 41 without a diabetes diagnosis), 39 individuals with chronic pancreatitis (19 with diagnosed diabetes and 20 without diagnosed diabetes), 20 individuals with long-standing (>3 years post-diagnosis) T2DM, 18 individuals with new-onset (≤3 years post-diagnosis) T2DM and 20 healthy subjects. To evaluate the influence of jaundice on biomarker performance, all Set 3 PDAC cases were further subcategorised as having low bilirubin levels (<20 mmol/L; upper level of normal for our Centre) or high bilirubin levels (>20 mmol/L). In Sets 1-3, individuals with PDAC had resectable disease and surgery was undertaken with curative intent, individuals with CP had histologically confirmed disease, and in all cases diabetes mellitus status was either participant-reported or determined via clinical measurement. Set 4 (n=71; Supplementary Table S3) a pre-diagnostic set used to determine whether biomarkers were altered prior to PDAC diagnosis, consisted of serum from women recruited to the UKCTOCS study who subsequently developed PDAC (n=35) and time- and centre-matched healthy controls (n=36).^16^ Samples were obtained up to 12 months prior to PDAC diagnosis and split into training and validation sets.

**Table 1:** Characteristics of the Set 2 (Training) and Set 3 (Validation) study cohorts.

a	Set 2 (n= 137)						
	PDAC	PDAC-DM	CP	CP-DM	LSDM	Healthy	
**Number**	41	41	10	10	20	15	
**Sex**							
Male	20	25	6	8	11	8	
Female	21	16	4	2	9	7	
**Age, years**							
Median	71	70	46.5	55	66.5	56	
IQR	(62-76)	(65-75)	(44-62.5)	(47.5-63.5)	(60.5-71)	(53-60)	
**CA19-9, U/mL**							
Median	86.9	154.6	10.1	17.1	15.4	11.5	
IQR	(32.0-290.7)	(44.2-336.3)	(6.1-17.1)	(5.6-49.6)	(10.5-23.5)	(8.2-14.0)	
**Bilirubin, U/mL**							
Median	22	21	5	8	-	-	
IQR	(9-41)	(8-50.5)	(4-6)	(7.3-9.5)	-	-	

b	Set 3 (n= 175)						
	PDAC	PDAC-DM	CP	CP-DM	LSDM	NOD	Healthy
**Number**	41	37	20	19	20	18	20
**Sex**							
Male	22	24	12	9	12	10	8
Female	19	13	8	10	8	8	12
**Age, years**							
Median	68	71.5	49.5	52	67.5	63	53
IQR	(60-73)	(63.75.)	(40.5-56)	(46.5-54.5)	(57.5-74)	(58.5-66)	(50-67)
**CA19-9, U/mL**							
Median	181.4	271.1	15.8	24.4	47.3	19.1	10.0
IQR	(70.8-375)	(87.7-426.4)	(8.9-49.2)	(15.3-31.4)	(16.8-61.4)	(10.0-31.2)	(7.0-17.6)
**Bilirubin, U/mL**							
Median	12	10	4	3.5	-	-	-
IQR	(8-39.5)	(6.5-31.5)	(3-6.5)	(3-5)	-	-	-
**BMI, kg/m^2^**							
Median	25.5	24.2	21.9	21.7	31.6	28.4	24.9
IQR	(22.2-27.5)	(22.6-27.8)	(19.8-24.3)	(19.1-24.3)	(29.8-37.0)	(23.9-36.5)	(24.3-30.0)
**AJCC Stage**							
IA	1	3	**-**	**-**	**-**	**-**	**-**
IB	-	-	**-**	**-**	**-**	**-**	**-**
IIA	2	8	**-**	**-**	**-**	**-**	**-**
IIB	33	15	**-**	**-**	**-**	**-**	**-**
III	-	-	**-**	**-**	**-**	**-**	**-**
IV	3	5	**-**	**-**	**-**	**-**	**-**

PDAC, pancreatic ductal adenocarcinoma; PDAC-DM, pancreatic cancer-associated diabetes; CP, chronic pancreatitis; CP-DM, chronic pancreatitis associated diabetes, DM, long-standing diabetes (>3yr post-diagnosis of DM); NOD, new-onset diabetes (<3yr post-diagnosis of DM); BMI, Body Mass Index; AJCC, American Joint Committee on Cancer.

### Sample collection

Glycated haemoglobin (HbA1c) was measured in blood collected in Sarstedt Monovette Fluoride/EDTA tubes (Sarstedt Ltd, Leicester, UK) by the RLUH Clinical Biochemistry Department. All other blood samples were collected in Sarstedt Monovette Serum Z tubes or K+EDTA tubes (Sarstedt Ltd, Leicester, UK) and allowed to stand for 30 minutes at room temperature before centrifugation at 800 × g for 10 minutes for serum fractionation and 16000 × g for 1 minute for plasma fractionation. Serum and plasma fractions were aliquoted into cryotubes and stored at −80°C. UKCTOCS blood samples were collected in Greiner gel tubes (Greiner Bio-one 455071) and transported at ambient temperature to a central laboratory, and processed within 20 hours of venipuncture. Samples were subjected to centrifugation at 4,000 rpm for 10 minutes and serum aliquoted and stored at -80°C.^17^

### Biomarker measurement and data filtering

Serum and/or plasma levels of adiponectin and IL-1Ra were measured using Luminex assays (Bio-Plex Pro Diabetes Adiponectin Assay; Catalogue no. 171A7003M and Bio-Plex Pro Human Cytokine 27-Plex Assay; Catalogue no. M500KCAFOY, respectively; Bio-Rad, UK) on a Bio-Plex 200 System (Bio-Rad, UK). All remaining analytes were measured using a Multiskan FC microplate photometer (ThermoFisher, MA, USA) using the following commercially available ELISA kits: Adrenomedullin (Human) EIA kit (Catalogue no. EK-010-01CE, Phoenix Pharmaceuticals, CA, USA); Alpha 1 antichymotrypsin Human ELISA kit (Catalogue no. ab157706, Abcam, Cambridge, UK); Apolipoprotein A1 Human ELISA kit (Catalogue no. ab108804, Abcam); Chemerin Human ELISA kit (Catalogue no. ab155430, Abcam); Human Clusterin (Apolipoprotein J) ELISA kit (Catalogue no. ab174447, Abcam); Human SPARC ELISA kit (Catalogue no. ab220654, Abcam); Transferrin Human ELISA kit (Catalogue no. ab108911, Abcam); Human Thrombospondin-1 Quantikine ELISA (Catalogue no. DTSP10, R&D Systems, MN, USA); VonWillebrand Factor (VWF) Human ELISA kit (Catalogue no. ab108918, Abcam); Pancreatic & GI Cancer (Mucin PC/CA199) ELISA kit (Catalogue no. 1840, Alpha Diagnostics, TX, USA). All samples were measured in duplicate, without blinding, following manufacturers’ instructions with inter-plate variability assessed using 3 quality controls per plate. To minimise bias, samples were randomised across plates. Biomarker concentrations were determined from standard curves of positive control proteins using four- or five-parameter logistic regression models. Inter-plate variation of ≤15% was considered acceptable. Any biomarkers with concentrations falling outside of the linear range and those with duplicate measurements having a coefficient of variance (CV) >20%, were removed from the dataset.

### Bioinformatics and statistical analyses

The pathway analyses were generated using Ingenuity Pathway Analysis (QIAGEN Inc., https://www.qiagenbioinformatics.com/products/ingenuity-pathway-analysis, RRID:SCR_008653). A Core Analysis was performed to identify the top canonical pathways, along with a diseases and functions analysis, of differentially expressed proteins between PDAC and healthy controls (HC). Enriched categories of canonical pathways were considered statistically significant if the negative log likelihood enrichment score exceeded 1.3 (p<0.05) using Fisher's exact test. A Biomarker Filter was performed to identify the most promising biomarker candidates significantly enriched for an association to diabetes.

JMP Version 14 (Statistical Analysis System Institute Inc., NC, USA, RRID:SCR_008567) and R version 3.6.2 (R Project for Statistical Computing, Vienna, Austria, RRID:SCR_001905) were used for statistical analysis and data processing. Protein expression data was analysed using a two-tailed Mann–Whitney U test, adjusted for multiple comparisons with the Holm-Bonferroni method, and a logistic regression model was used to select the most promising marker combinations. Multivariable models were considered adjusting for age, BMI and serum CA19-9 concentration. P-values were calculated for each model predictor using a Wald test. To assess the diagnostic accuracy of each candidate marker, predicted probabilities from the logistic regression were used to construct Receiver Operating Characteristic (ROC) curves, with Area Under the Curve (AUC) estimated using the trapezoidal rule, and 95% confidence intervals for AUC calculated using the DeLong method. A two-sided significance level of p-values less than 0.05 was used throughout. Spearman's rank correlation coefficient (r_s_(df)) was used to assess the strength of the linear relationship between adiponectin and BMI, where df are the degrees of freedom.

### Role of funders

The funders played no role in the study design, the collection, analysis, or interpretation of data, nor the preparation, or approval of the manuscript for publication.

## Results

### Multi-stage process for discovery and validation of protein biomarkers for early detection of PDAC

**Figure 1:**
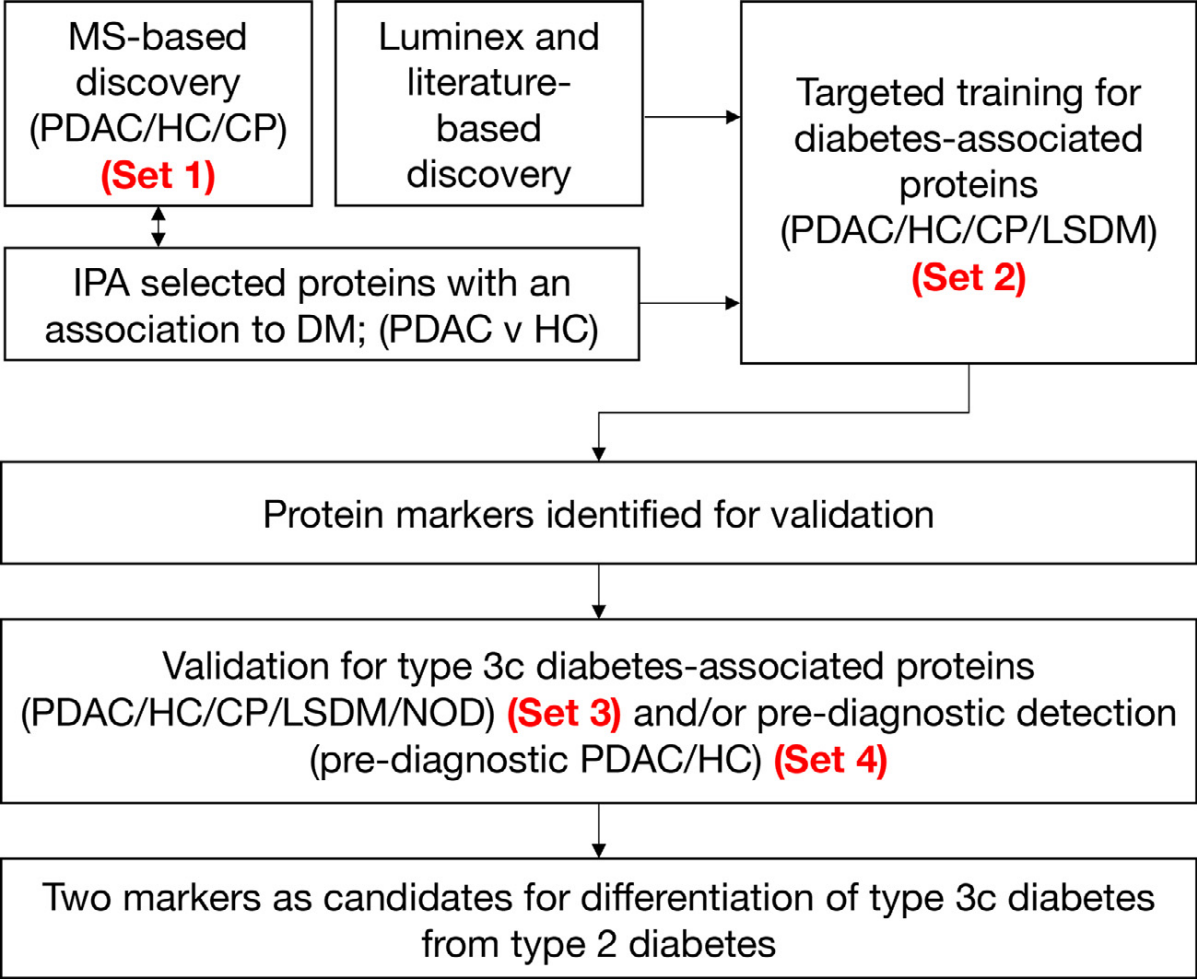
Biomarker development pathway. Biomarkers were selected via a multi-stage process using up to four independent sample sets. MS, mass spectrometry; PDAC, pancreatic cancer; CP, chronic pancreatitis; LSDM, long-standing diabetes (>3yr post-diagnosis of DM); NOD, new-onset diabetes (<3 yr post-diagnosis of DM).

We previously reported mass spectrometry-based (isobaric tags for relative and absolute quantification /iTRAQ) for blood-borne protein biomarkers that distinguished PDAC from healthy controls (HC), individuals with chronic pancreatitis (CP), or obstructive jaundice (Figure 1).^16^^,^^18^^,^^19^ Functional analysis of differentially expressed proteins identified in discovery, with results filtered by disease categories and functions, revealed an enrichment in proteins associated with glucose metabolism disorder (p=1.19×10-13), metabolism of protein (p=1.11×10-12), and diabetes mellitus (p=7.59×10-12) (Table 2). Application of a biomarker filter, prioritising for proteins associated with diabetes, provided a list candidate proteins for biomarker development. Additionally, of 27 cytokines previously tested in Luminex experiments, five significantly altered in PDAC compared to HC^20^ were highlighted for development (Figure 1). Of markers emerging from MS- and Luminex-based discovery, together with promising markers identified within literature,^21^, ^22^, ^23^, ^24^, ^25^, ^26^ a total of 30 markers were selected for development. A nested set (Set 1, Supplementary Table S1) of a larger retrospective cohort described previously,^16^ was used to observe analyte levels in healthy individuals versus individuals with histologically confirmed PDAC with- and without diagnosed diabetes. Subsequently we undertook new targeted training and validation for up to twelve diabetes-associated proteins in sets containing samples from individuals with long-standing diabetes mellitus (LSDM; Set 2; Table 1a) and NOD (Set 3; Table 1b). Where appropriate, candidate biomarkers were finally validated in a pre-diagnostic cohort (Set 4; Supplementary Table S2^16^).

**Table 2:** Mass spectrometry-detected proteins and associated DM-related pathways identified by IPA enriched in PDAC.

Pathway	P value	Genes coding for identified proteins
Glucose metabolism disorder	1.19×10^−13^	ADIPOQ,AGT,AHSG,ALB,AMBP,APOB,APOC3,APOD, APOE,APOM,C3,C4A/C4B,C5,CFB,CFD,CLEC3B,CLU, CRP,F10,F2,FCGR3A/FCGR3B,GPLD1,HBB,HP,LBP, PON1,PPIA,RBP4,SERPINC1,SERPIND1,SERPINF1, SHBG,TF,THBS1,TNXB
Metabolism of protein	1.11×10^−12^	AFM,AGT,AHSG,ALB,APCS,APOA2,APOA4,APOB, APOE,C1S,C3,C4A/C4B,C4BPA,CLU,CP,CST3,F2,F5, FGA,GPLD1,GSN,IGFALS,IGFBP3,ITIH2,KLKB1,KNG1,PROC,SAA1,SERPINA1,SERPINA10,SERPINC1, SERPIND1,TF,THBS1
Diabetes mellitus	7.59×10^−12^	ADIPOQ,AGT,AHSG,ALB,AMBP,APOB,APOC3,APOD, APOE,APOM,C3,C4A/C4B,C5,CFB,CLEC3B,CLU, CRP,F10,FCGR3A/FCGR3B,GPLD1,HBB,HP,PON1,PPIA,RBP4,SERPINC1,SERPIND1,SERPINF1,SHBG,TF,TNXB

DM, diabetes mellitus; IPA, Ingenuity Pathway Analysis.

In total, 264 proteins were considered at the discovery stage, and up to 30 proteins were taken through training (Set 2) and validation (Set 3). Eighteen candidates showed an increased level in PDAC compared to diabetes (LSDM or NOD) and 8 showed a decreased level in PDAC (Table 3; Supplementary Figures S1 + S2). With only three exceptions, Apo-A1, C-Peptide and TSP-1, all candidates examined were similarly altered in individuals with PDAC regardless of DM status. Two candidates, SPARC and transferrin failed to significantly distinguish PDAC from LSDM. Glucagon, PAI-1 and TSP-1 distinguished PDAC-DM from LSDM, but not from NOD. In total, 12 analytes showed significantly different levels between PDAC-DM and both LSDM and NOD, with AUCs for the distinction of PDAC-DM from LSDM and NOD ranging from 0.48 to 0.96 (Supplementary Tables S4 and S5, respectively). A logistic regression model was used to select the markers which performed best in combination. After pairs of markers exhibiting strong correlations (Supplementary Figure S3) were removed, amongst the potential biomarkers with the best combined performance for discriminating PDAC-DM from DM were adiponectin and IL-1Ra.

**Table 3:** Candidate biomarkers evaluated by immunoassays in training and/or validation sample sets.

	P value (Set 2)		P value (Set 3)	Δ
Protein	PDAC v PDAC-DM	PDAC-DM v LSDM	PDAC-DM v NOD	
Adiponectin^§†^	ns	0.001	0.001	Up
Adrenomedullin^§‡^	ns	0.004	nd	Up
Alpha 1-antichymotrypsin^§†^	ns	0.01	nd	Up
Apo-A1^†^	0.02	ns*	nd	Up
Chemerin^§‡^	ns	ns*	nd	-
Clusterin^†^	ns	0.01	nd	Down
C-Peptide^†^	0.05	0.0002	0.009	Up
Ghrelin^†^	ns	0.04	<0.0001	Down
GIP^†^	ns	<0.0001	<0.0001	Down
GLP-1^†^	ns	0.002	ns	Down
Glucagon^†^	ns	0.01	ns	Down
IFN-G^‡^	ns	0.02	ns*	Up
IL-1Ra^‡^	ns	0.03	<0.0001	Up
IL-4^‡^	ns	0.008	0.0002	Up
IL-6^‡^	ns	0.004	0.03	Up
IL-7^‡^	ns	0.03	nd	Up
IL-8^‡^	ns	<0.0001	0.007	Up
IL-9^‡^	ns	0.0005	0.03	Up
IL-12^‡^	ns	0.03	0.03	Up
Insulin^†^	0.002	<0.0001	ns*	Down
Leptin^§†^	ns	0.0002	ns*	Down
MIP-1A^‡^	ns	0.009	ns*	Up
MIP-1B^‡^	ns	<0.0001	0.002	Up
PAI-1^†^	ns	0.01	ns	Up
PDGF-BB^‡^	ns	0.02	0.02	Up
RANTES	ns	nd	0.001	Up
SPARC^†^	ns	ns	nd	-
Transferrin^†^	ns	ns	nd	-
TSP-1	0.04	0.01	ns*	Down
VWF^†^	ns	<0.0001	nd	Up

PDAC, pancreatic ductal adenocarcinoma; PDAC-DM, pancreatic cancer-associated diabetes; DM, long-standing diabetes (>3yr post-diagnosis of DM); NOD, new-onset diabetes (<3yr post-diagnosis of DM); Δ = the direction of change in analyte concentration from diabetes (LSDM or NOD) to PDAC, § = measured in both serum and plasma, † = data shown are from serum, ‡ data shown are from plasma, ns = non-significant, *denotes that significance was not established for the comparison shown, however, was established for the following comparison: PDAC vs DM p<0.05, nd = not determined, ns = not significant (p>0.05). p-values calculated using the Mann-Whitney U test.

### Adiponectin as a candidate marker for early PDAC detection

**Figure 2:**
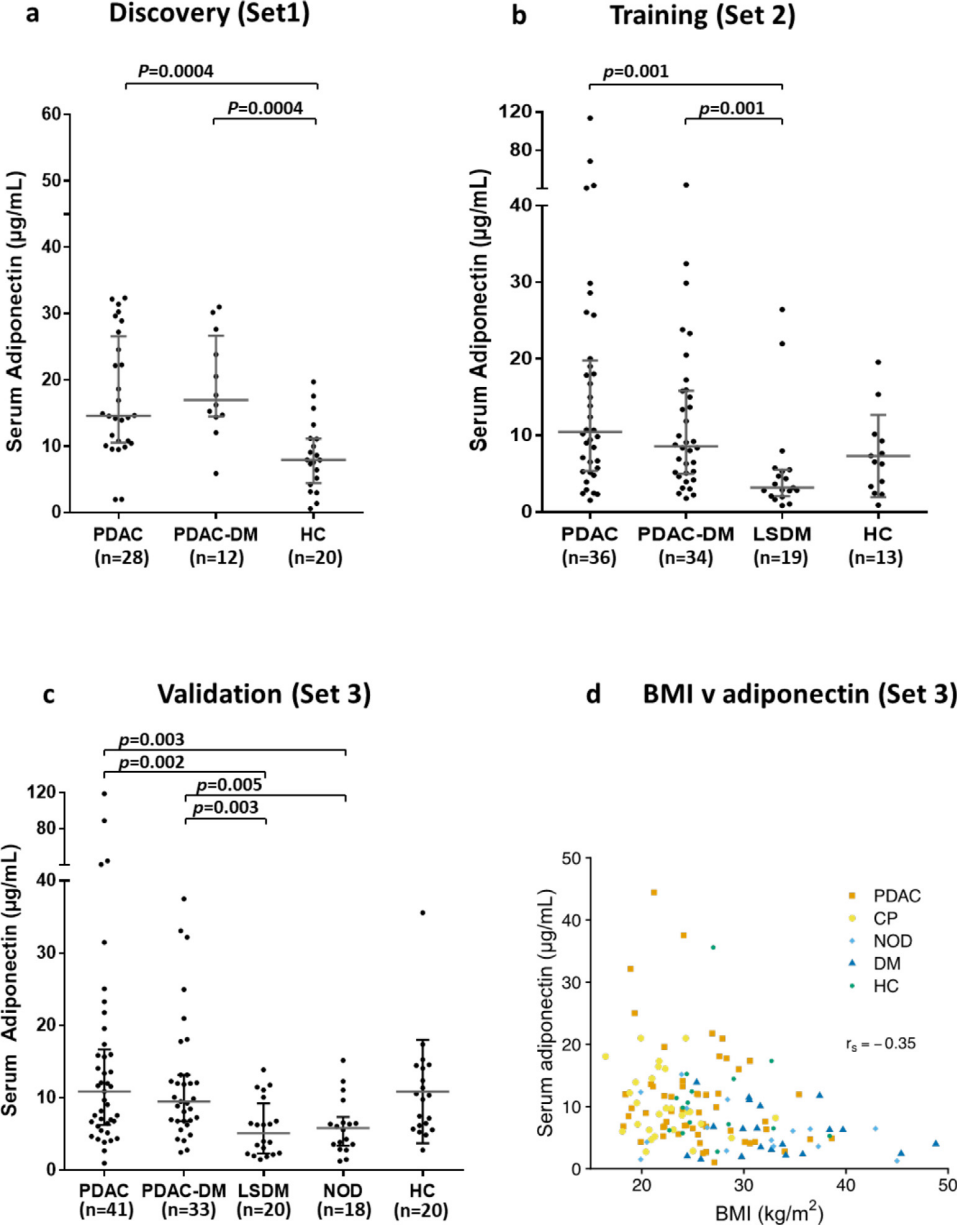
Serum adiponectin measured in three independent diagnostic cohorts. Serum levels of adiponectin are unchanged in PDAC regardless of diabetes status (a) and are elevated in PDAC and PDAC-DM compared to long-standing diabetes (b) and new-onset T2DM (NOD) (c). Adiponectin shows a moderate but significant negative correlation with BMI across all sample groups, with (rs(143)= -0.359, p<0.0001 (d). The median and interquartile range are shown for each group. p-values were calculated using the Mann–Whitney U test, adjusted for multiple comparisons with the Holm-Bonferroni method. PDAC, pancreatic cancer; PDAC-DM, pancreatic cancer-related diabetes; LSDM, long-standing diabetes (>3yr post-diagnosis of DM); NOD, new-onset diabetes (<3yr post-diagnosis of DM).

Circulating adiponectin levels are lower in individuals with T2DM compared to healthy subjects.^27^ We, however, observed no such reduction in serum adiponectin when DM was associated with PDAC (Figure 2a). In training samples adiponectin levels in individuals with PDAC with and without DM were significantly higher than in individuals with LSDM (p=0.001, p=0.001, Figure 2b). Validation in an independent cohort (Set 3; Figure 2c) confirmed that significantly elevated levels of adiponectin were present in individuals with PDAC with and without DM, compared to those with LSDM (p=0.003, p=0.002) or NOD (p=0.005, p=0.003). We previously demonstrated that obstructive jaundice can alter the circulating levels of some proteins.^28^ In both Set 2 and Set 3 a trend in elevation of adiponectin levels was observed in PDAC cases with high serum bilirubin (>20 μmol/L; the upper level of normal for our Centre; p=0.07 and p=0.06, respectively). Nevertheless, when samples from patients with high bilirubin were removed from the analysis, the elevation in adiponectin levels in PDAC cases with and without DM, compared to those with LSDM and NOD remained significant (ranging from p=0.009 to p=0.0004), indicating that adiponectin levels discriminate between PDAC and DM independently of obstructive jaundice.

Adiponectin negatively correlates with Body Mass Index (BMI).^29^^,^^30^ In Set 3, which contained representation from all of the groups in the study (Table 1b), BMI levels were highest in the LSDM and NOD groups and lowest in the CP group. Median BMI was significantly higher in DM versus PDAC, CP and HC (p<0.0001, p<0.0001, and p=0.01, respectively), and in PDAC versus CP (p=0.0001), but did not differ between PDAC and HC. Although adiponectin showed a moderate but significant negative correlation with BMI across all sample groups (r_s_(143)= -0.359, p<0.0001; Figure 2d), differences in BMI alone could not account for adiponectin levels. For example, despite significantly higher BMI in PDAC versus CP, median adiponectin levels were indistinguishable between PDAC (+/- DM) and CP (+/- DM) groups, respectively. Taken together, our data suggest that normal or raised serum adiponectin in individuals newly diagnosed with DM could indicate T3cDM. Validation of adiponectin as a marker of PDAC in this setting is merited.

### IL-1Ra as a candidate marker for early PDAC detection

**Figure 3:**
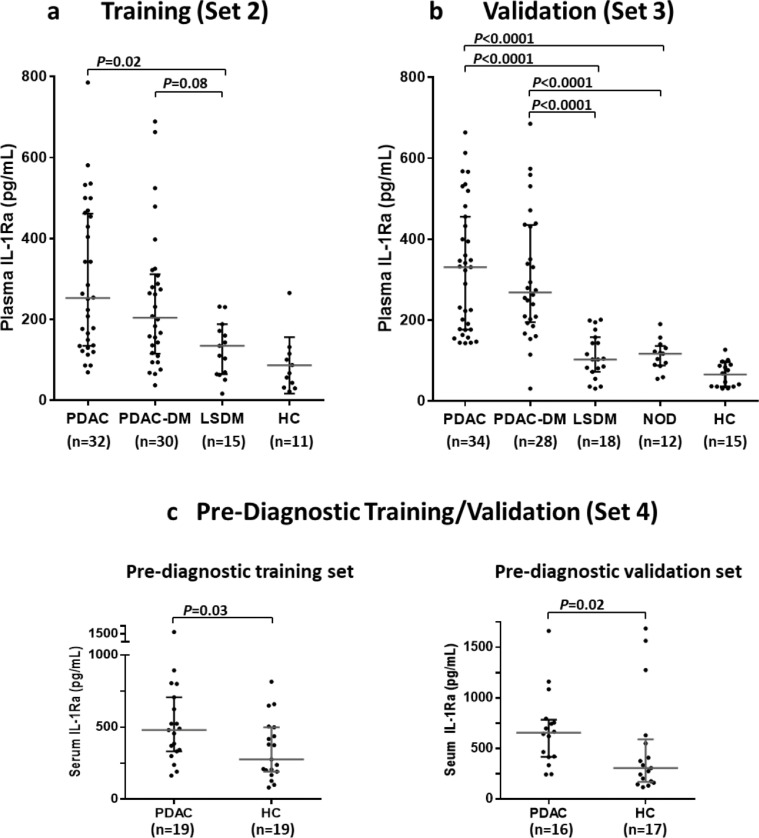
Plasma and serum IL-1Ra measured in four independent cohorts. IL-1Ra is upregulated in patients with PDAC and PDAC-DM compared to those with T2DM, regardless of DM duration (a, b). Upregulation of IL-1Ra is observed up to 12 months prior to diagnosis as shown in pre-diagnostic training and validation serum samples (c). The median and interquartile range are shown for each group. p-values were calculated using the Mann–Whitney U test, adjusted for multiple comparisons with the Holm-Bonferroni method. PDAC, Pancreatic cancer; PDAC-DM, pancreatic cancer-related diabetes; LSDM, long-standing diabetes mellitus; NOD, new-onset diabetes mellitus; HC, healthy controls.

Elevation in circulating levels of IL-1Ra is associated with insulin resistance and T2DM.^31^ Consistent with this we observed elevated plasma levels of IL-1Ra in both LSDM and NOD compared to healthy controls (Figure 3a and b). Previously we showed that serum IL-1Ra levels were unaffected by jaundice and elevated in PDAC compared to healthy controls (p<0.05).^20^ Measurement of plasma IL-1Ra in independent training samples (Set 2) supported this observation (p<0.0001), with plasma levels further shown to be elevated in individuals with both PDAC and PDAC-DM compared to individuals with LSDM (p=0.02 and p=0.08, respectively; Figure 3a). The elevation in IL-1Ra in PDAC and PDAC-DM individuals, compared to LSDM, was validated in Set 3 (p<0.0001; Figure 3b) and shown also to be true compared to individuals with NOD (p<0.0001; Figure 3b). To examine the potential of IL-1Ra as an early marker for PDAC, PDAC pre-diagnostic serum samples from the UK Collaborative Trial of Ovarian Cancer Screening (UKCTOCS) study^32^ (Set 4) were analysed. In two independent subsets of pre-diagnostic UKCTOCS samples, IL-1Ra showed a significant upregulation up to 12 months prior to PDAC diagnosis (p=0.03 and 0.02; Figure 3b). Our data support the validation of circulating IL-1Ra as a potentially valuable marker for earlier detection of PDAC in high-risk individuals newly diagnosed with DM.

### Adiponectin and IL-1Ra distinguish type 3c diabetes from type 2 diabetes

**Figure 4:**
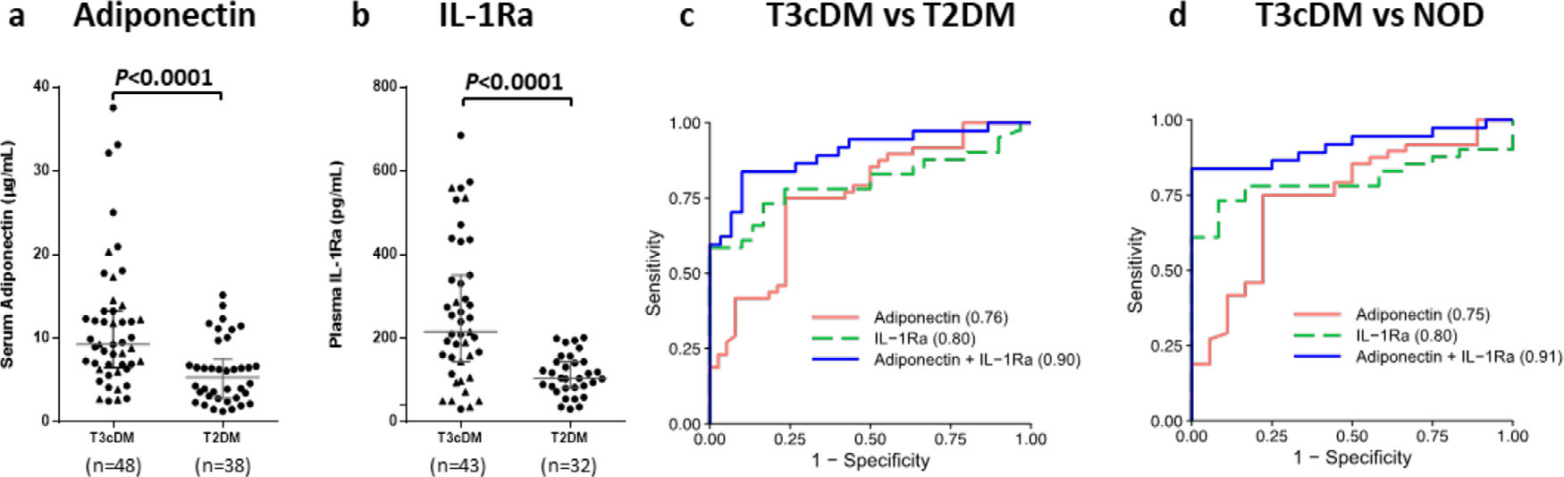
Blood levels of adiponectin and IL-1Ra measured in T3cDM and T2DM individuals (Set 3) and associated receiver operator characteristic (ROC) curve analyses. Serum levels of adiponectin (a) and plasma levels of IL-1Ra (b) are significantly elevated in patients with T3cDM (●=PDAC- and ▲=CP-related) compared to those with T2DM (adiponectin: n=33 PDAC-DM, n=15 CP-DM, IL-1Ra: n=28 PDAC-DM, n=15 CP-DM). In combination, serum adiponectin and plasma IL-1Ra achieved an AUC of 0.90 (95% confidence interval (CI): 0.83-0.97) in distinguishing T3cDM from T2DM (n=37 and 30, respectively), with an optimal sensitivity of 83.7% (CI: 68.0-93.8%) and a specificity of 90.0% (CI: 73.5-97.9%) (c). In the distinction of T3cDM from among individuals with NOD (n=37 and 12, respectively), the combined markers achieved an AUC of 0.91 (CI: 0.84-0.99) with optimal sensitivity and specificity of 83.7% (CI: 64.9-92.0%) and 100.0% (CI: 73.6-100.0%), respectively (d). For A and B, the median and interquartile range are shown for each group. T3cDM, type 3c diabetes mellitus; T2DM, type 2 diabetes mellitus; NOD, new-onset diabetes mellitus; AUC, area under the curve.

Using samples from individuals with both PDAC- and CP-related DM, we explored whether blood levels of adiponectin and IL-1Ra could serve to identify T3cDM among individuals newly diagnosed with T2DM. Both biomarkers were found to be independent of one another (rs(142)= 0.0569, p<0.0001) with a significant separation in median biomarker levels observed between the two diabetes subtypes (p<0.0001; Figure 4a and b). The combination of adiponectin with IL-1Ra achieved an Area Under the Receiver Operating Characteristic Curve (AUC) of 0.90 (95% confidence interval (CI): 0.83-0.97) in distinguishing T3cDM (PDAC- and CP-related) from T2DM, with an optimal sensitivity of 83.7% (CI: 68.0-93.8%) and specificity of 90.0% (CI: 73.5-97.9%) (Figure 4c). More specifically, in the distinction of T3cDM from individuals with NOD, the combination of adiponectin and IL-1Ra achieved an AUC of 0.91 (CI: 0.84-0.99) with optimal sensitivity and specificity of 83.7% (CI: 64.9-92.0%) and 100.0% (CI: 73.6-100.0%), respectively (Figure 4d). Serum adiponectin levels have been shown to correlate positively with age.^33^ Significant moderate positive correlations between age and adiponectin levels were observed in NOD (rs(17)= 0.50, p=0.03) and LSDM (rs(19)=0.46, p=0.04), but not in PDAC or PDAC-DM. The median age in the CP-DM group (52 yr) was lower than in the PDAC-DM group (71.5 yr), reflecting the earlier age of onset of CP compared to PDAC. To test whether age, or BMI confounded the discriminatory power of adiponectin and IL-1Ra in distinguishing T3cDM from T2DM or NOD they were incorporated into the model (Supplementary Table S6 and S7, respectively). While this generated an improvement in performance (vs T2DM AUC = 0.96 [CI: 0.91-1.00], vs NOD AUC = 0.93 [CI: 0.86-1.00] compared to 0.90 and 0.91 respectively), both adiponectin (vs T2DM OR = 1.27, CI: 1.05-1.71; vs NOD 1.31, CI: 1.03-1.93) and IL-1Ra (vs T2DM OR = 1.03, CI: 1.01-1.05; vs NOD OR = 1.03, CI: 1.01-1.06) retained their association with T3cDM.

### CA19-9 is elevated in type 2 diabetes

**Figure 5:**
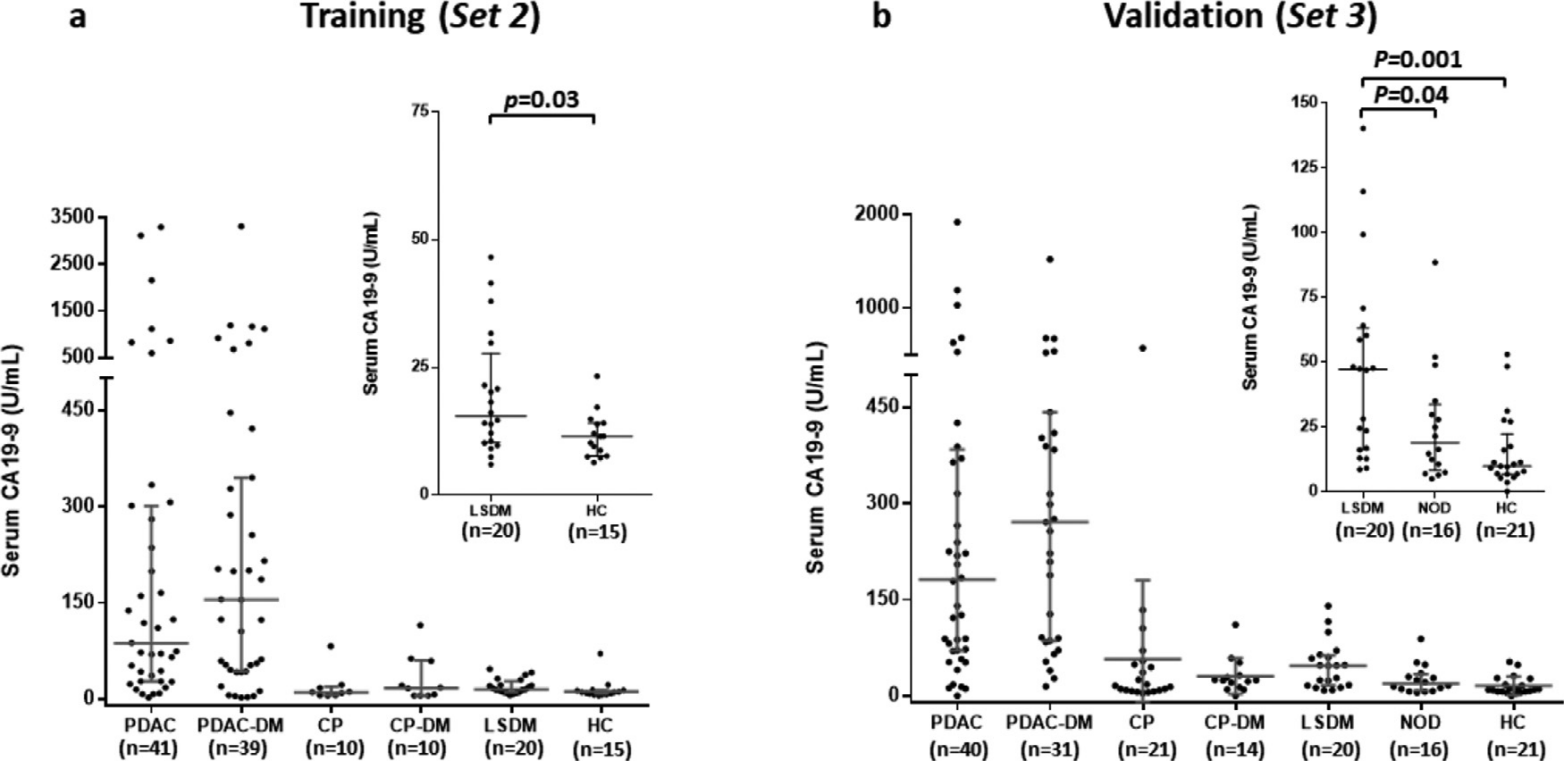
Serum CA19-9 measured in two independent diagnostic cohorts. In training (a) and validation (b) (S*ets 2* and *3*) serum CA19-9 levels were significantly elevated in PDAC, regardless of DM status, compared to all other controls (p<0.0001). No difference in CA19-9 level was observed between PDAC-DM and PDAC. In non-cancer controls, CA19-9 was significantly elevated in LSDM compared to healthy controls (p=0.03 and p=0.001 in *Sets 2* and *3*, respectively), however, no significant upregulation was observed between NOD and HC. For a and b, the median and interquartile range are shown for each group. PDAC, pancreatic cancer; PDAC-DM, pancreatic cancer-related diabetes; CP, chronic pancreatitis; CP-DM, chronic pancreatitis-related diabetes; LSDM, long-standing diabetes (>3yr post-diagnosis of DM); NOD, new-onset diabetes (<3yr post-diagnosis of DM).

Circulating CA19-9 is the only biomarker routinely used in the clinical management of PDAC^34^ and the performance of novel biomarkers is often compared with that of CA19-9. Elevation of CA19-9 in PDAC-related DM has led to its suggested use as a PDAC screening aid in individuals with NOD.^35^^,^^36^ However, CA19-9 is also elevated in benign diseases, including DM.^37^^,^^38^ Here we found that CA19-9 levels were significantly elevated in PDAC, regardless of DM status, compared to all other controls (both p<0.0001; Figure 5a and b). No difference in CA19-9 level was observed between PDAC-DM and PDAC. In non-cancer controls, CA19-9 was significantly elevated in LSDM compared to healthy controls (p=0.03 and p=0.001; Sets 2 and 3, respectively). Interestingly, a significant elevation in CA19-9 levels was observed between LSDM and NOD (p=0.04), but not between NOD and HC (Figure 5b).

The addition of CA19-9 to the IL1-Ra + adiponectin panel did not significantly alter its performance. For the distinction of T3cDM v T2DM, IL1-Ra + adiponectin + CA19-9 achieved an AUC=0.91 (CI: 0.83-0.97) compared to AUC=0.90 (CI: 0.83-0.98) for IL1-Ra + adiponectin alone. For the distinction of T3cDM v NOD, IL1-Ra + adiponectin + CA19-9 achieved an AUC=0.92 (CI: 0.84-0.99) versus AUC=0.91 (CI: 0.84-0.99) for the two-marker panel.

## Discussion

Through the evaluation of biomarkers to distinguish T3cDM from T2DM, this study aimed to provide a means of enriching the NOD population for PDAC, with a view to facilitating earlier PDAC detection through screening. We noted that the behaviour of adiponectin in T2DM was at odds with its behaviour in PDAC-related DM. Adiponectin is synthesised and secreted predominantly by adipocytes into peripheral blood. T2DM and insulin resistance are associated with low circulating adiponectin (hypoadiponectinemia, <4 μg/mL).^39^ However, in PDAC, regardless of DM status, we found that adiponectin levels were either similar to- or modestly higher than in healthy subjects. Why plasma adiponectin levels do not fall in PDAC-DM is unclear, although it may relate to PDAC-associated metabolic changes. Circulating adiponectin concentrations are negatively correlated with both subcutaneous adipose tissue (SAT) and visceral adipose tissue (VAT).^29^^,^^40^ Moreover, reductions in body weight, regardless of diabetes status are accompanied by increases in circulating adiponectin.^27^ While we found that differences in BMI alone could not account for adiponectin levels in the groups we studied, data on weight loss in PDAC patients were unavailable. Others^41^ have shown that individuals with PDAC undergo several metabolic and soft tissue changes in the months preceding PDAC diagnosis including decreases in serum lipids, body weight, and SAT and ultimately in VAT, and muscle. Levels of the browning marker, uncoupling protein 1 (UCP1) are elevated in SAT from individuals with PDAC.^41^ UCP1 mRNA in SAT is positively correlated with both serum adiponectin and adiponectin mRNA levels in SAT.^42^ Understanding whether adiponectin plays a biological role in the metabolic processes occurring in PDAC will require more research.

IL-1Ra, an anti-inflammatory protein secreted by various types of cells including immune cells, epithelial cells, and adipocytes, reduces the endogenous activity of the IL-1 family of pro-inflammatory cytokines, primarily via competitive inhibition of the IL-1R1 receptor.^43^ Circulating IL-1Ra is increased in obesity and T2DM and correlates with insulin resistance,^31^ while intracellular β-cell-derived IL-1Ra is downregulated in T2DM leading to IL-1β-induced β-cell dysfunction and apoptosis.^44^^,^^45^ In-line with these findings we confirmed increased levels of circulating IL-1Ra in T2DM compared to healthy controls and further showed that IL-1Ra levels are elevated in PDAC compared to both LSDM and NOD. This supports IL-1Ra as a candidate to enrich for PDAC amongst NOD. The significant upregulation in IL-1Ra up to 12 months prior to PDAC diagnosis further indicate its potential as an early biomarker of PDAC.

Until suitable cohorts are available, the lack of pre-diagnostic samples from PDAC patients, accurately annotated for NOD, prevents assessment of the utility of CA19-9 for early PDAC detection among individuals with NOD. Here, the addition of CA19-9 to the IL1-Ra and adiponectin panel did not improve its performance in distinguishing T3cDM from T2DM. However, a role for CA19-9 as part of a panel for early detection of PDAC in individuals with NOD merits testing. The performance of CA19-9 to distinguish pre-diagnostic PDAC cases from controls has been evaluated and shown to hold discriminatory power which improves significantly as patients approach diagnosis.^46^^,^^47^ As CA19-9 is raised in the majority of PDAC patients at- or after diagnosis, and as our sample cohorts, with the exception of Set 4, included only diagnosed PDAC individuals, CA19-9 was not included alongside adiponectin and IL-1Ra in the present study. The slight elevation of CA19-9 in DM is, however, noteworthy, and needs consideration in future assessment of CA19-9 for inclusion in biomarker panels to distinguish T2DM from T3cDM.

An important limitation when interpreting our findings is that three of the four cohorts utilised were obtained from a single centre. Although Set 4 was obtained from a multi-centre UK-wide study, future independent validation of adiponectin and IL-1Ra will be required. Further to this, test data confirming when individuals were recently clinically normoglycemic prior to DM diagnosis were not available to this study. Consequently, some individuals categorised as NOD may have had DM for >3 yr prior to sample donation. Since adiponectin and IL1-Ra were elevated in PDAC regardless of DM status and behaved similarly in control individuals with either long-standing or new-onset DM, knowledge of the duration of diabetes was not essential here. However, we recognise that diabetes duration affects PDAC risk and is an important consideration for future studies. Additional limitations of this study include the lack of complete DM data in the UKCTOCS cohort. Adiponectin's strength in the context of this study lies in its ability to stratify DM into PDAC-DM and T2DM with no robust separation between PDAC and HC. The performance of adiponectin could not therefore be meaningfully evaluated in the UKCTOCS cohort, which consisted of samples from individuals with PDAC and HC with incomplete diabetes data. Finally, our study was conducted using only PDAC cases undergoing surgery, validation of findings in advanced PDAC patients is necessary.

Whilst biomarker development here focused on PDAC, biomarkers specific for T3cDM could have the added benefit of also detecting individuals with T3cDM secondary to CP. There are pre-diagnostic NOD cohorts currently open to recruitment in the US, led by the Consortium for the Study of Chronic Pancreatitis, Diabetes and Pancreatic Cancer (CPDPC)^48^ and in the UK (the NOD cohort of the UK-Early Detection Initiative for pancreatic cancer, UK-EDI).^2^^,^^49^ These cohorts will offer the best chances of validating the biomarkers presented here and other published biomarkers, in the stratification of individuals newly diagnosed with T2DM for PDAC screening.

## Data sharing statement

The data generated and analysed during this study are described in the following data record: https://doi.org/10.7910/DVN/9FMMJI. All code used in the preparation of this manuscript is available on GitHub at: https://github.com/antshevans/Oldfield-type3c-diabetes-biomarker-paper.git, archived at: https://doi.org/10.5281/zenodo.5764055. The study protocols can be obtained via request to the corresponding author.

## Funding

This work was supported by grants from North West Cancer Research, UK (CR1142), Cancer Research UK (C7690/A26881, C18616/A25153) and Pancreatic Cancer Action, UK.

## Contributors

EC, WG and LO study concept and design. EP advised on statistics and data analysis. UM, JT, WG, CH, PG and TP advised on patient selection and provided samples. LO, CJ and RR performed assays. LO and AE analysed and verified data and prepared figures and tables. LO and EC drafted the manuscript. All authors provided critical revision of the manuscript and approved the final version.

## Declaration of Competing Interest

LO, EC, WG, CH and PG are named as inventors on GB patent GB1806002.0; PCT/GB2019/050998, submitted by the University of Liverpool, that covers the measurement of adiponectin and IL-1Ra as a biomarker for early detection of pancreatic cancer. UM holds patent number EP EP10178345.4 for breast cancer diagnosis, and has stock ownership awarded by the University College London (UCL) in Abcodia.

## Appendix Supplementary materials

Image, application 1
