# Supplementary material for: Blood levels of adiponectin and IL-1Ra distinguish type 3c from type 2 diabetes: Implications for earlier pancreatic cancer detection in new-onset diabetes
Source: eBioMedicine. 2022 Jan 3;75:103802. doi: 10.1016/j.ebiom.2021.103802 (PMC8741427; doi:10.1016/j.ebiom.2021.103802)
Supplement: Supplementary file 1 [file mmc1.pdf]

Supplementary Figure S1

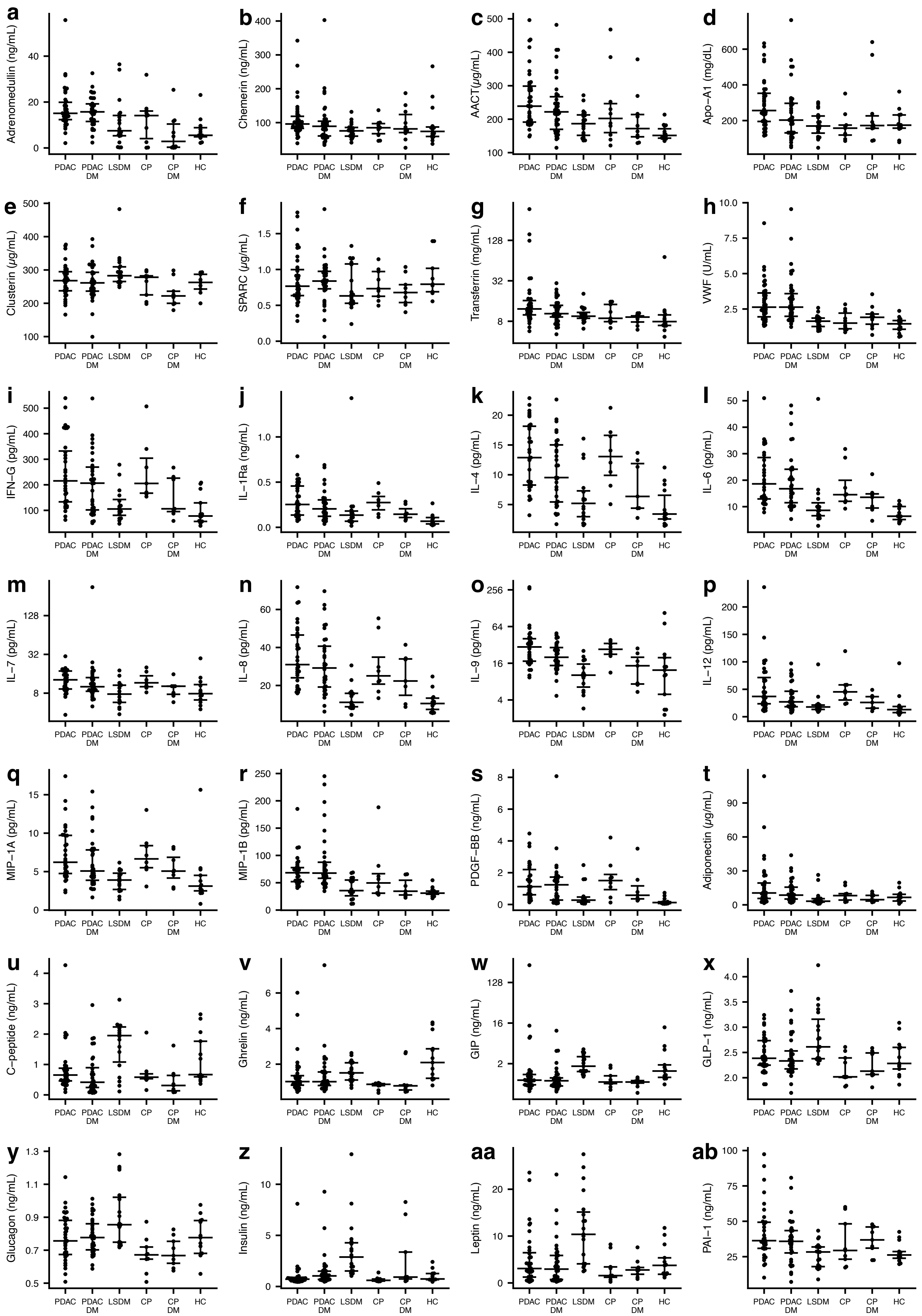

Supplementary Figure S1. Candidate biomarkers evaluated by immunoassays in Set 2, listed in Table 3. Analytes measured in plasma by ELISA (**a-b**) and Luminex (**i-s**) are shown, alongside those measured in serum by ELISA (**c-h**) and Luminex (**t-ab**).

Supplementary Figure S2

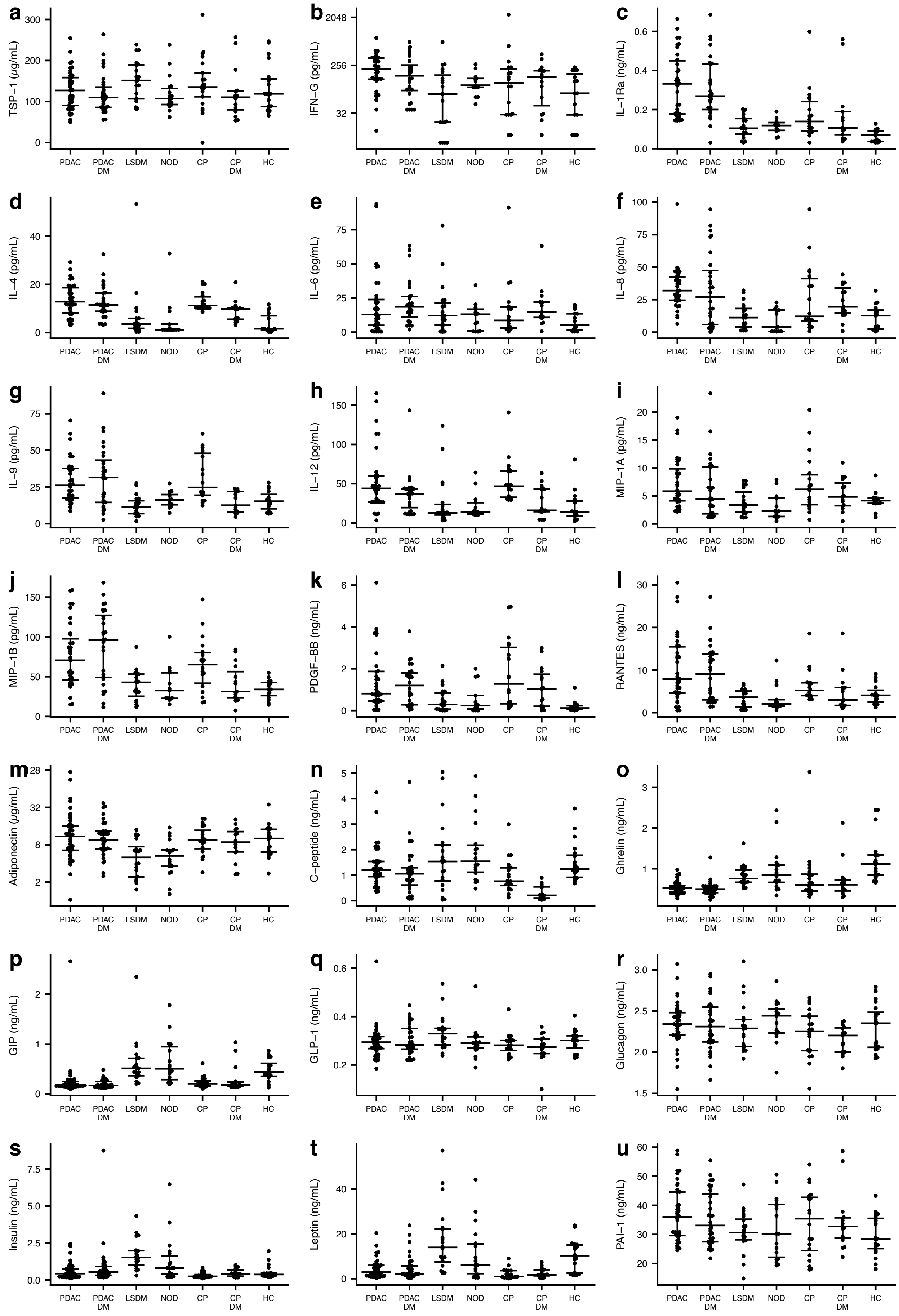

Supplementary Figure S2. Candidate biomarkers evaluated by immunoassays in Set 3, listed in Table 3. Analytes measured in serum by ELISA (**a**) and Luminex (**m-u**) are shown, as are those measured in plasma using Luminex (**b-l**).

Supplementary Figure S3.

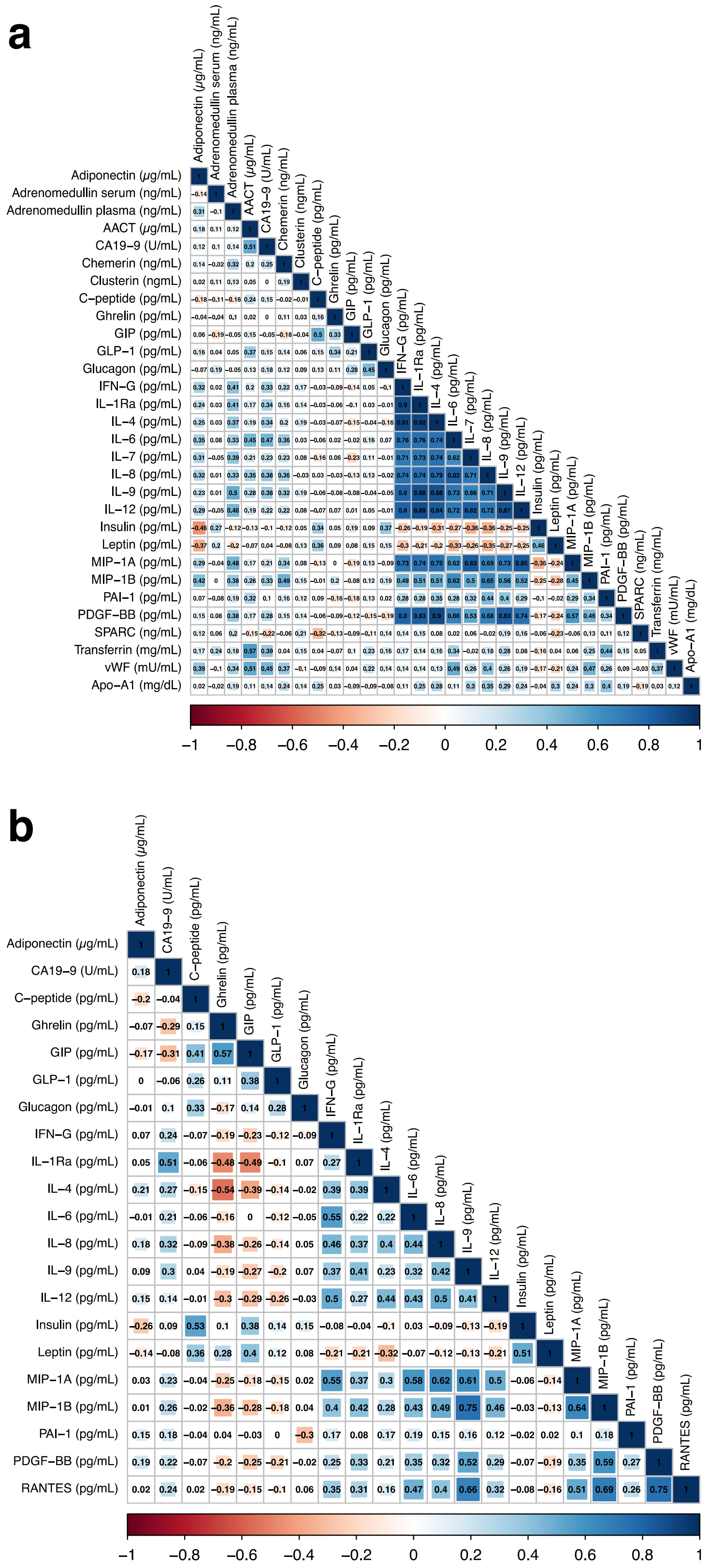

Supplementary Figure S3. Spearman's rank correlation matrices for candidate markers in Set 2 (**a**) and Set 3 (**b**).

Supplementary Table S1. Characteristics of the Set 1 (Discovery) study cohort

	**Set 1 (n=60)**		
	PDAC	PDAC-DM	Healthy
**Number**	28	12	20
**Sex**			
Male	17	4	15
Female	11	8	5
**Age, years**			
Median	67	69	33.5
IQR	(60-72)	(62.5– 73.5)	(28-37)
**CA19-9, U/mL**			
Median	127.4	83.8	3.2
IQR	(44.4-127.4)	(20.7-192.4)	(1.6-4.6)

PDAC, pancreatic ductal adenocarcinoma; PDAC-DM, pancreatic cancer-associated diabetes

Supplementary Table S2. Classification of pancreatic cancer cases in Set 3 according to TNM stage

		PDAC	PDAC-DM
**T**	**1**	2	3
	**2**	0	0
	**3**	34	24
**N**	**0**	3	11
	**1**	33	16
**M**	**0**	36	27
	**1**	3	5

PDAC, pancreatic ductal adenocarcinoma; PDAC-DM, pancreatic cancer-associated diabetes

Supplementary Table S3. Characteristics of the pre-diagnostic UKCTOCS cohort (Set 4)

	**Set 4 (n=71)**			
	Training		Validation	
	PDAC cases	Controls	PDAC cases	Controls
**Number**	19	19	16	17
**Sex**				
Male	0	0	0	0
Female	19	19	16	17
**Age**, years				
Median	70	62	67	59
IQR	(60-71.5)	(56.5-69)	(62.5-72.5)	(55-63)
**CA19-9, U/mL**				
Median	37.6	4.3	35.4	11.1
IQR	(5.4-83.1)	(0.2-21.6)	(10.5-98.4)	(8.0-18.0)
**BMI, kg/m^2**				
Median	24.4	27.0	26.2	24.8
IQR	(23.0-28.8)	(22.9-29.4)	(24.2-31.6)	(23.1-27.1)

PDAC, pancreatic ductal adenocarcinoma; PDAC-DM, pancreatic cancer-associated diabetes; IQR, interquartile range; BMI, Body Mass Index.

Supplementary Table S4. ROC analyses of candidate markers evaluated in Set 2 (training) comparing PDAC-DM with LSDM cases.

	PDAC-DM vs LSDM				
	AUC (95% CI)	Sensitivity at 99% specificity	Sensitivity at 97.5% specificity	Sensitivity at 95% specificity	Sensitivity at 90% specificity
AACT (μg/mL)	0.69 (0.56-0.82)	23.8%	23.8%	28.6%	50.0%
Adiponectin (μg/mL)	0.78 (0.64-0.92)	8.8%	8.8%	8.8%	14.7%
Adrenomedullin plasma (ng/mL)	0.74 (0.58-0.9)	0.0%	0.0%	0.0%	0.0%
Adrenomedullin serum (ng/mL)	0.63 (0.43-0.83)	6.2%	6.2%	6.2%	15.6%
Apo-A1 (mg/dL)	0.59 (0.44-0.74)	25.0%	25.0%	25.0%	33.3%
C-peptide (pg/mL)	0.82 (0.69-0.95)	15.6%	15.6%	15.6%	37.5%
CA19-9 (U/mL)	0.88 (0.78-0.98)	75.0%	75.0%	86.1%	86.1%
Chemerin (ng/mL)	0.61 (0.47-0.76)	15.0%	15.0%	22.5%	27.5%
Clusterin (ngmL)	0.71 (0.57-0.84)	41.2%	41.2%	41.2%	44.1%
Ghrelin (pg/mL)	0.67 (0.52-0.82)	14.7%	14.7%	14.7%	26.5%
GIP (pg/mL)	0.87 (0.77-0.97)	76.5%	76.5%	76.5%	76.5%
GLP-1 (pg/mL)	0.76 (0.63-0.88)	41.2%	41.2%	41.2%	52.9%
Glucagon (pg/mL)	0.71 (0.57-0.86)	29.4%	29.4%	29.4%	32.4%
IFN-G (pg/mL)	0.7 (0.55-0.85)	22.6%	22.6%	22.6%	32.3%
IL-12 (pg/mL)	0.7 (0.54-0.87)	3.2%	3.2%	3.2%	38.7%
IL-1Ra (pg/mL)	0.69 (0.52-0.86)	0.0%	0.0%	0.0%	43.3%
IL-4 (pg/mL)	0.74 (0.59-0.89)	16.1%	16.1%	16.1%	35.5%
IL-6 (pg/mL)	0.82 (0.67-0.97)	0.0%	0.0%	0.0%	51.5%
IL-7 (pg/mL)	0.7 (0.52-0.87)	9.7%	9.7%	9.7%	22.6%
IL-8 (pg/mL)	0.87 (0.77-0.98)	45.9%	46.6%	47.7%	63.6%
IL-9 (pg/mL)	0.82 (0.69-0.95)	38.7%	38.7%	38.7%	48.4%
Insulin (pg/mL)	0.85 (0.74-0.95)	50.0%	50.0%	50.0%	67.6%
Leptin (pg/mL)	0.81 (0.69-0.93)	44.1%	44.1%	44.1%	50.0%
MIP-1A (pg/mL)	0.73 (0.59-0.87)	38.2%	38.2%	38.2%	44.1%
MIP-1B (pg/mL)	0.85 (0.74-0.96)	48.5%	48.5%	48.5%	48.5%
PAI-1 (pg/mL)	0.72 (0.58-0.86)	27.3%	27.3%	27.3%	39.4%
PDGF-BB (pg/mL)	0.71 (0.55-0.88)	6.2%	6.2%	6.2%	34.4%
SPARC (ng/mL)	0.59 (0.42-0.77)	2.9%	2.9%	8.8%	8.8%
Transferrin (mg/mL)	0.62 (0.47-0.76)	10.5%	10.5%	31.6%	36.8%
vWF (mU/mL)	0.87 (0.78-0.96)	51.3%	51.3%	61.5%	74.4%

AUC, Area under the curve; **PDAC-DM**, pancreatic cancer-associated diabetes; **LSDM**, long-standing diabetes (>3yr post-diagnosis of DM)

Supplementary Table S5. ROC analyses of candidate markers evaluated in Set 3 (validation) comparing PDAC-DM with NOD cases.

	PDAC-DM vs NOD				
	AUC (95% CI)	Sensitivity at 99% specificity	Sensitivity at 97.5% specificity	Sensitivity at 95% specificity	Sensitivity at 90% specificity
Adiponectin (μg/mL)	0.77 (0.63-0.91)	21.2%	21.2%	21.2%	29.7%
C-peptide (pg/mL)	0.72 (0.58-0.87)	21.2%	21.2%	21.2%	30.3%
CA19-9 (U/mL)	0.96 (0.92-1)	75.8%	75.8%	75.8%	90.9%
Ghrelin (pg/mL)	0.85 (0.72-0.98)	9.1%	9.1%	9.1%	42.4%
GIP (pg/mL)	0.91 (0.84-0.99)	66.7%	66.7%	66.7%	69.7%
GLP-1 (pg/mL)	0.48 (0.32-0.65)	0.0%	0.0%	0.0%	12.1%
Glucagon (pg/mL)	0.58 (0.41-0.74)	3.0%	3.0%	3.0%	18.2%
IFN-G (pg/mL)	0.6 (0.42-0.77)	25.0%	25.0%	25.0%	35.7%
IL-12 (pg/mL)	0.71 (0.52-0.9)	3.6%	3.6%	3.6%	10.7%
IL-1Ra (pg/mL)	0.93 (0.84-1)	78.6%	78.6%	78.6%	89.3%
IL-4 (pg/mL)	0.87 (0.71-1)	0.0%	0.0%	0.0%	64.3%
IL-6 (pg/mL)	0.72 (0.55-0.89)	17.9%	17.9%	17.9%	39.3%
IL-8 (pg/mL)	0.77 (0.62-0.91)	53.6%	53.6%	53.6%	53.6%
IL-9 (pg/mL)	0.72 (0.56-0.87)	57.1%	57.1%	57.1%	57.1%
Insulin (pg/mL)	0.61 (0.43-0.78)	6.1%	6.1%	6.1%	12.1%
Leptin (pg/mL)	0.66 (0.48-0.83)	6.1%	6.1%	6.1%	6.1%
MIP-1A (pg/mL)	0.67 (0.5-0.84)	32.1%	32.1%	32.1%	32.1%
MIP-1B (pg/mL)	0.8 (0.67-0.94)	46.4%	46.4%	46.4%	64.3%
PAI-1 (pg/mL)	0.62 (0.44-0.8)	3.0%	3.0%	3.0%	12.1%
PDGF-BB (pg/mL)	0.73 (0.56-0.9)	17.9%	17.9%	17.9%	35.7%
RANTES (pg/mL)	0.81 (0.67-0.95)	39.3%	39.3%	39.3%	53.6%
TSP-1 (μg/mL)	0.49 (0.32-0.65)	3.0%	3.0%	3.0%	12.1%

AUC, Area under the curve; **PDAC-DM**, pancreatic cancer-associated diabetes; **NOD**, new-onset diabetes (<3yr post-diagnosis of DM)

Supplementary Table S6. Multivariable logistic regression analysis predicting type 3c from type 2 diabetes (longstanding and new-onset), comparing models with adiponectin and IL1-Ra alone plus the addition of age and BMI.

	**Analytes alone**			**Analytes + age + BMI**		
	Odds Ratio	*95% CI*	*p*	*Odds Ratio*	*95% CI*	*p*
Intercept	0.00	0.00 – 0.04	**0.001**	25.35	0.01 – 81919.90	0.410
Adiponectin (∝g/mL)	1.37	1.14 – 1.75	**0.003**	1.27	1.05 – 1.71	**0.043**
IL-1Ra (pg/mL)	1.02	1.01 – 1.04	**0.006**	1.03	1.01 – 1.05	**0.016**
Age				0.95	0.85 – 1.05	0.330
BMI				0.80	0.64 – 0.94	**0.020**

n = 57

Supplementary Table S7. Multivariable logistic regression analysis predicting type 3c from new-onset type 2 diabetes, comparing models with adiponectin and IL1-Ra alone plus the addition of age and BMI.

	**Analytes alone**			**Analytes + age + BMI**		
	Odds Ratio	*95% CI*	*p*	*Odds Ratio*	*95% CI*	*p*
Intercept	0.00	0.00 – 0.19	**0.024**	1.79	0.00 – 16751.98	0.896
Adiponectin (∝g/mL)	1.34	1.08 – 1.85	**0.029**	1.31	1.03 – 1.93	0.075
IL-1Ra (pg/mL)	1.02	1.01 – 1.05	**0.028**	1.03	1.01 – 1.06	**0.040**
Age				0.93	0.79 – 1.06	0.331
BMI				0.94	0.75 – 1.16	0.579

n = 39
